# Supplementary material for: Biochemical and biophysical characterisation of immunoglobulin free light chains derived from an initially unbiased population of patients with light chain disease
Source: PeerJ. 2020 Mar 17;8:e8771. doi: 10.7717/peerj.8771 (PMC7083161; doi:10.7717/peerj.8771)
Supplement: Supplemental Information 1 [file peerj-08-8771-s001.docx]

**­­­­­Supplementary Materials: Biochemical and biophysical characterisation of immunoglobulin free light chains derived from an initially unbiased population of patients with light chain disease**

Rebecca Sternke-Hoffmann^1^, Amelie Boquoi^2^, David Lopez^2^, Florian Platten^3^, Roland Fenk^2^, Rainer Haas^2,*^ and Alexander K. Buell^1,4,*^

*^1^Institute of Physical Biology, Heinrich-Heine-University Düsseldorf*

*^2^Department of Hematology, Oncology and Clinical Oncology*

*Heinrich-Heine-University Düsseldorf*

*^3^Condensed Matter Physics Laboratory, Heinrich-Heine-University Düsseldorf*

*^4^Present address: Department of Biotechnology and Biomedicine, Technical University of Denmark, Lyngby, Denmark*

**To whom correspondence should be addressed:* [*haas@med.uni-duesseldorf.de*](mailto:haas@med.uni-duesseldorf.de) *and* [*alebu@dtu.dk*](mailto:alebu@dtu.dk)

**Supplementary Methods**

**Sample preparation**

As first step, the protein content of a 24 h urine collection was precipitated by ammonium sulfate (70% saturation), generally on the same day when the urine was obtained from the patient. After incubation at 4°C under continuous agitation, the samples were centrifuged at 6000 xg for 25 min at 4°C. The precipitates were dissolved in 10 mM phosphate buffer, pH 7.5, and dialyzed against the same buffer at 4°C for 72 h (the buffer was exchanged every 24 h). The light chains were purified by size-exclusion chromatography on an ÄKTA pure chromatography system (GE Healthcare) using a Superdex 75 10/300 GL column equilibrated in the same buffer.

The final protein concentration was estimated by the absorbance at 280 nm. The used extinction coefficient (38.000 cm-1 M-1) was estimated from a range of published light chain sequences and corresponds to an average composition of 3.5 Tryptophan and 9 Tyrosin residues.

**Analysis of the LC and HSA-ratio**

In order to characterize the purity of the samples with respect to contamination with human serum albumin, Western blots were performed. Before (in most cases) or after the purification (P002, P014) by a size-exclusion chromatography step, the samples were diluted by a denaturing, non-reducing loading buffer and run on an SDS-PAGE gel. The separated proteins were transferred to a membrane and detected either by an antibody against anti-human kappa light chain (BioLegend), against anti-human lambda light chain (BioLegend) or anti-human serum albumin (Santa Cruz Biotechnology). The antibodies were used at a concentration of 0.5 µg/ml and detected by a secondary anti-mouse antibody.

By correlating the bands of the SDS-PAGE gel with these blots, the relative amount of LC’s compared to the HSA-content could be estimated using the program ImageJ, by integrating the degree of Coomassie staining. For this analysis the monomer and dimer band of both proteins were considered but no bigger aggregates.

**Differential scanning calorimetry (DSC)**

Thermal unfolding of the various light chains was studied using a MicroCal VP-DSC instrument (Malvern, UK) by performing temperature ramps on the LC solutions at an estimated concentration of 25 µM (based on the UV extinction coefficient mentioned above) from 10°C to 90°C with a heating rate of 0.8°C/min. For each sample, the unfolding temperature, Tm, was estimated from the peak of the thermogram. The reversibility of the unfolding was examined by a second heating of the samples after cooling down to 10°C. The degree of refolding was estimated by the ratio of the areas under the unfolding peaks of the second to the first temperature scan.

**Determination of the dimer content**

To determine the ratio of dimers to monomers of the various LC’s, the proteins were analyzed by running the samples before purification via SEC on a denaturing, non-reducing SDS-PAGE gel and staining the gels with Coomassie blue. The gels were parsed photometrically using the program ImageJ. For this analysis only the identified monomer and dimer bands were used, no larger and smaller bands.

**Proteolysis**

LCs were incubated at 37°C in 10 mM phosphate buffer, pH 7.5 with 1 M urea at an estimated concentration of 33 µM with bovine trypsin (molar ratio 1:100). The preparation was done on ice and the aliquot for the first time point was collected after trypsin addition and immediately inhibited by adding trypsin inhibitor (at an excess of 2:1). Further aliquots were taken 1, 2, 18, 24 and 48 h after the addition of the trypsin and quenched by the addition of trypsin inhibitor. The samples were analyzed by SDS-PAGE. Before running the samples on an SDS-PAGE gel the samples were diluted into a denaturing sample buffer without a reducing agent and heated at 95°C for 10 min.

The aggregation behavior in the presence of trypsin was examined by adding the fluorescent dye Thioflavin-T (ThT) and measurement of the fluorescence intensity (Excitation: 450 nm; Emission: 490 nm) in a multi-well plate reader (FLUOstar Omega or CLARIOstar, BMG labtech).

**Aggregation behavior**

Different solution conditions were tested for their potential to induce aggregation of the patient-derived, purified LCs. In order to examine the aggregation behavior of the LC’s at pH 4, different protein concentrations in a range from 5 µM to 50 µM were tested. The final samples were prepared by diluting protein solutions of different concentrations from 10 mM PB pH 7.4 1:1 into 300 mM citric acid buffer at pH 4. The experiment was performed in a multi-well plate reader (BMG labtech; FLUOstar Omega, CLARIOstar) at 37°C under mild shaking conditions (every 5 min for 15 s) in a multi-well, low binding plate. The amyloid formation was monitored using 20 µM of the fluorescent dye Thioflavin-T which binds to β-sheet rich structures and displays the binding with an increase of fluorescence intensity (Excitation: 450 nm; Emission: 490 nm). Subsequently the samples were analyzed using atomic-force microscopy (AFM, instruments by JPK and Bruker were used; cantilever: QMCL-AC16OTS) to confirm the presence of amyloid fibrils.

**Supplementary Data**

**Sample purity**

In order to characterize the purity of the samples and the relative amounts of LCs to human serum albumin (HSA) and other proteins, Western blots with anti-kappa, anti-lambda and anti-HSA antibodies were performed in addition to SDS-PAGE gels. The Western blots were used for identification, but not for quantification, because the binding of the antibody can be influenced by conformational changes, fragmentation or posttranslational modifications (1). Albuminuria is often associated with kidney damage by MM and the percentage of urinary albumin excretion obtained from 24-hour urine collection among patients with AL can range between 5 and 85% (average 70%) (2,3). Some of the examined samples contain a high amount of HSA and only a small amount of LCs (see Figure S1). The samples from P008 and P009 contained large amounts of HSA, while also containing relevant quantities of light chains. These quantities do not show any obvious correlation with the clinical category of the individual patients. However, it is worth noting that none of the samples derived from a patient in category 1, the least severe, contains much HSA, reflecting the intact filtration unit of the glomerulus. The remaining samples were all largely dominated (~60-90%) by light chains. Nevertheless, the findings of the samples P008 and P009 will not be included in the subsequent experimental results, because the HSA content had an influence on the measurements and can not be immaculately distinguished.


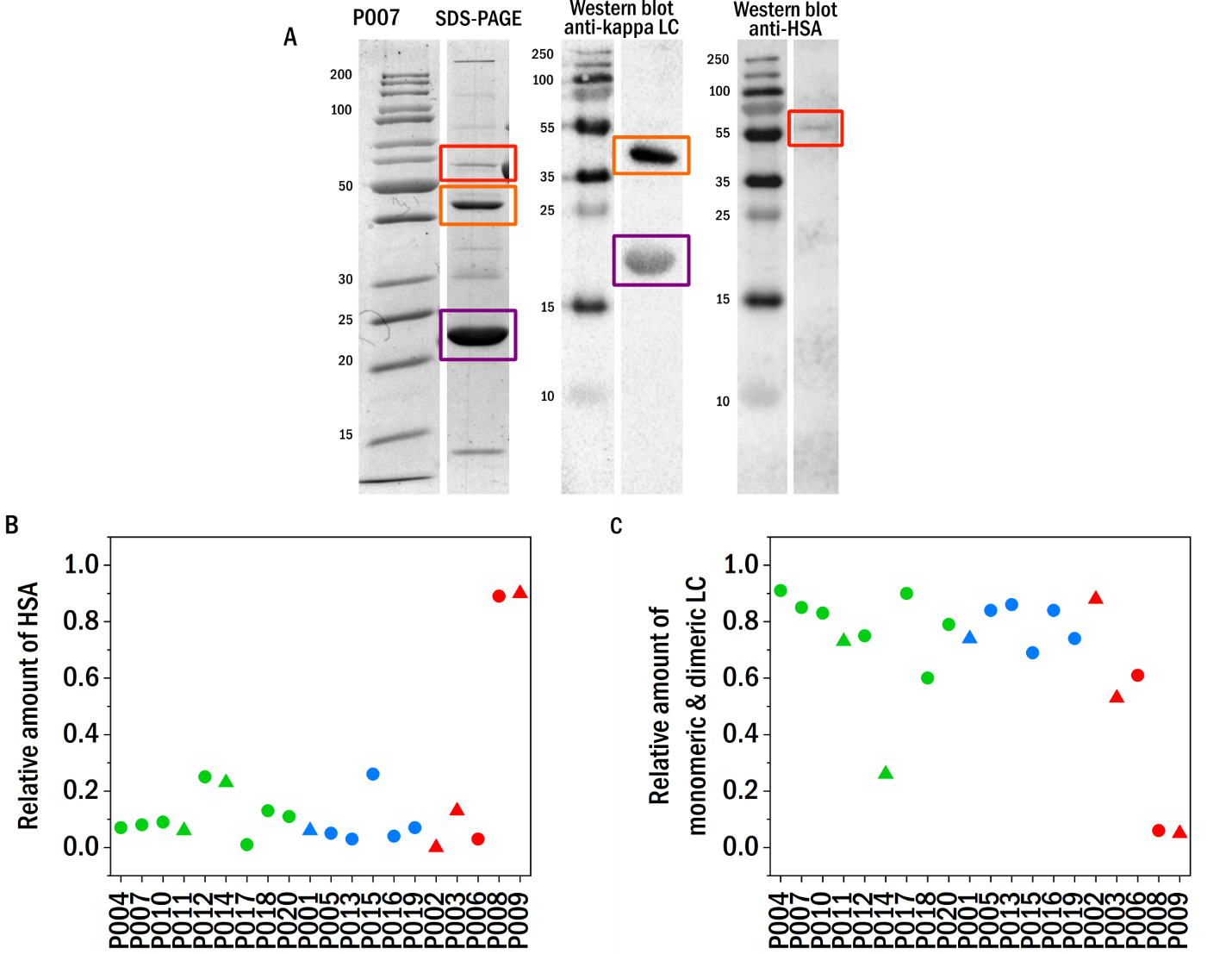


Figure S1: Sample purity estimates from SDS-PAGE gels and Western Blots. a) P007 as an example. The HAS-band is marked with a red square, the light chain-bands in orange (dimer) and purple (monomer). b) The relative amount of HSA in the different samples (left) b) The relative amount of light chain (monomer and dimer combined) in the different samples (right). The sorting and colors indicate the categories into which the patients were classified based on the severity of their renal impairment (CDK stage: 1+2 (green), 3 (blue), 4+5 (red). The shape symbolizes the isotype of the LC (triangle: lambda, circle: kappa).


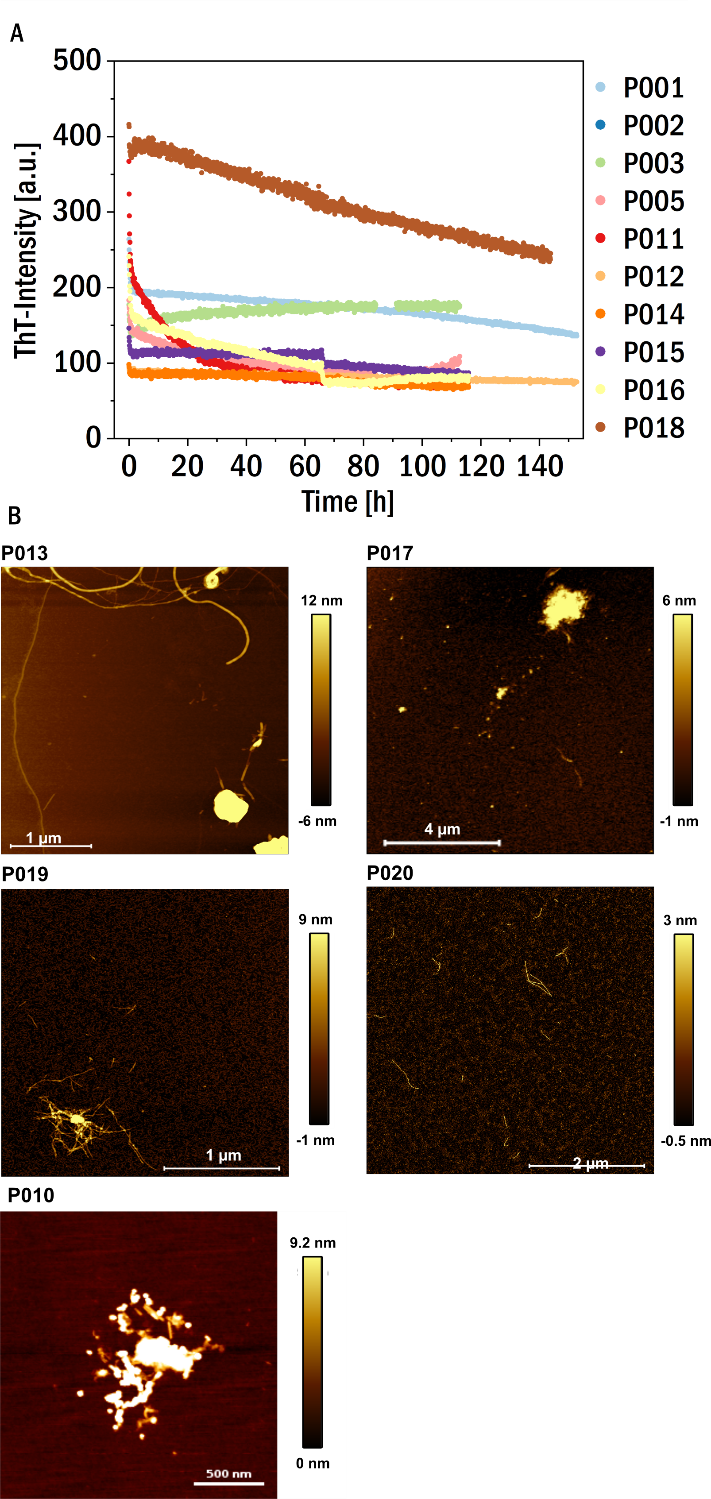


Figure S2: Aggregation of the LCs in the presence of trypsin. A) ThT-fluorescence aggregation assay of the non-amyloid forming LC in the presence of trypsin (P006 is already displayed in Figure 3 of the main manuscript). B) AFM-height images of the aggregated (ThT-positive) samples of P013, P017, P019, P020, P010.


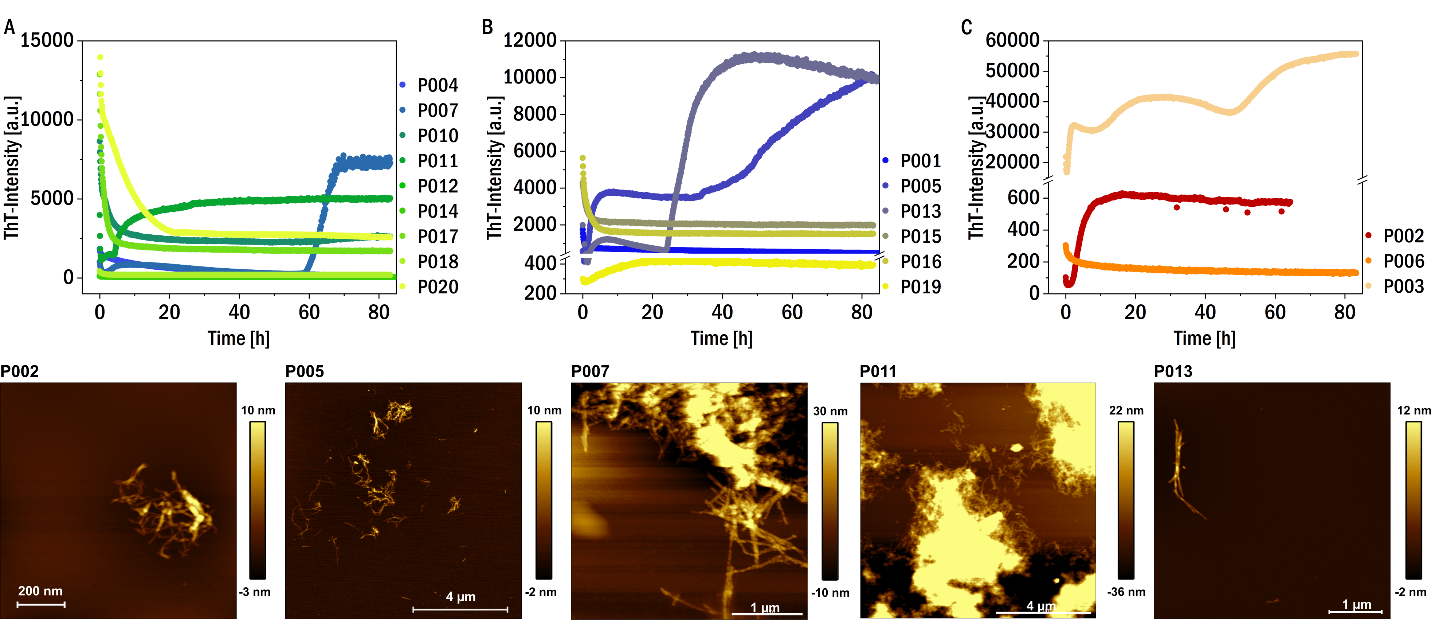


Figure S3: Aggregation assay at pH 4 in the absence of reducing agent. Top: ThT-fluorescence aggregation assays at pH 4 of the LCs of category I (A), II (B), III (C) corresponding to figure 4F. Bottom: AFM-height images of the aggregated samples of P002, P005, P007, P011 and P013.


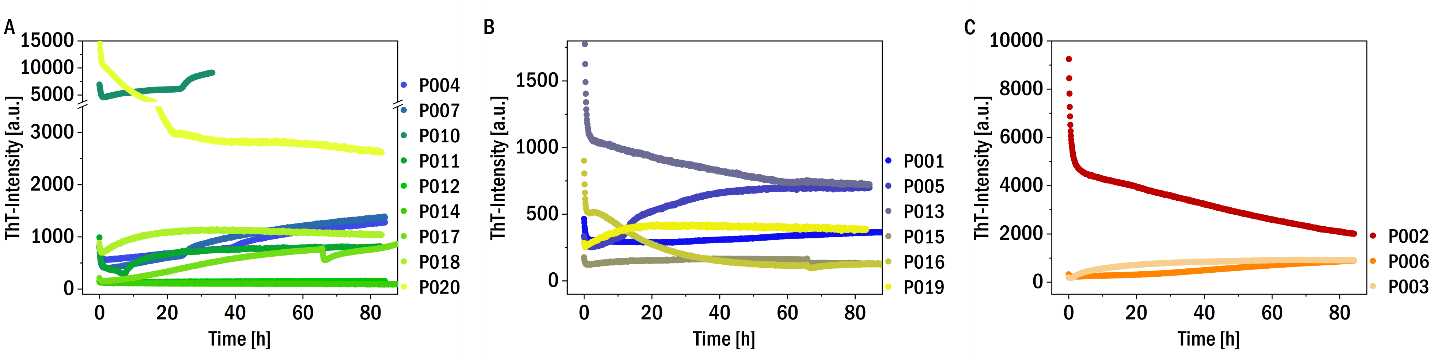


Figure S4: Aggregation at pH 7.5 in the presence of 1 mM TCEP. ThT-fluorescence aggregation assays at pH 7.4 in the presence of 1 mM TCEP of the LCs of category I (A), II (B), III (C).

We also tested for correlations between some of the biophysical and biochemical parameters (Figure S5). We find weak inverse correlation between thermal stability (as quantified by melting temperature) and on the one hand the ability to refold and on the other hand degradability by trypsin. In other words, there seems to a weak relationship in the sense that the more thermostable a LC, the smaller its ability to refold. And also, the more thermostable, the more resistant against proteolytic digestion by trypsin. A putative explanation for the first relationship is that a very hydrophobic core that stabilizes the native state against thermal unfolding can also lead to irreversible aggregation, once the LC is actually unfolded. The second relationship may be explained by the fact that more thermostable LCs are also less dynamic at room temperature and hence more difficult to degrade by trypsin.

These relationships between biophysical and biochemical parameters do not have any direct relevance for the main purpose of the study, which is the elucidation between LC properties and impairment of kidney function. We report them nevertheless for their general interest and relevance to protein science.


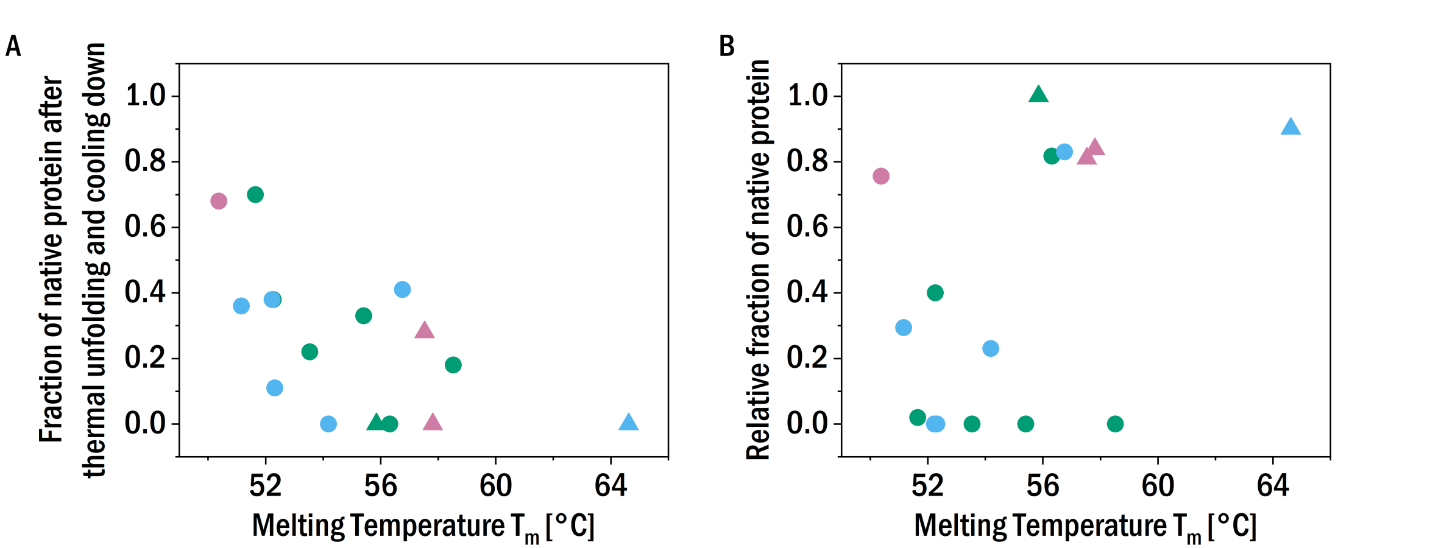


Figure S5: A) The fraction of native protein that unfolds during the second scan against the melting temperature Tm determined from the differential scanning calorimetry (DSC) experiments of the different samples allocated in the three patient categories according to renal impairment (CDK stage): I (green), II (blue), III (red). The correlation coefficient is -0.597 with a p-value of 0.015. B) The relative fraction of native protein (monomer and dimer combined) after 48 h incubation with trypsin against the melting temperature Tm determined from the differential scanning calorimetry (DSC) experiments of the different samples. The colors refer to the corresponding patient category defined above: I (green), II (blue), III (red). The correlation coefficient is 0.468 with a p-value of 0.067.

**Supplementary References**

1. Bass J, Wilkinson D, Rankin D, Phillips B, Szewczyk N, Smith K, et al. An overview of technical considerations for Western blotting applications to physiological research. Scand J Med Sci Sports. 2017 Jan;27(1):4–25.

2. Leung N, Gertz M, Kyle RA, Fervenza FC, Irazabal MV, Eirin A, et al. Urinary albumin excretion patterns of patients with cast nephropathy and other monoclonal gammopathy-related kidney diseases. Clin J Am Soc Nephrol. 2012 Dec;7(12):1964–8.

3. Sugihara H, Chihara D, Seike K, Fukumoto K, Fujisawaa M, Suehara Y, et al. Percentage of urinary albumin excretion and serum-free light-chain reduction are important determinants of renal response in myeloma patients with moderate to severe renal impairment. Blood Cancer J. 2014 Aug;4(8):e235.
